# Genotoxicity and Reproductive Risk in Workers Exposed to Pesticides in Rural Areas of Curicó, Chile: A Pilot Study

**DOI:** 10.3390/ijerph192416608

**Published:** 2022-12-10

**Authors:** Natalia Landeros, Soledad Duk, Carolina Márquez, Bárbara Inzunza, Ian S. Acuña-Rodríguez, Liliana A. Zúñiga-Venegas

**Affiliations:** 1 Centro Oncológico, Facultad de Medicina, Universidad de Católica del Maule, Talca 3466706, Chile; 2 Laboratorio de Investigaciones Biomédicas (LIB), Facultad de Medicina, Universidad de Católica del Maule, Talca 3466706, Chile; 3 Laboratorio de Citogenética y Genética Toxicológica, Facultad de Ciencias Biológicas, Universidad de Concepción, Concepción 4070386, Chile; 4 Instituto de Investigaciones Interdisciplinarias (I^3^), Universidad de Talca, Talca 3465548, Chile; 5 Centro de Investigación de Estudios Avanzados del Maule (CIEAM), Universidad Católica del Maule, Talca 3466706, Chile

**Keywords:** genotoxicity, reproductive risk, agricultural workers, pesticide exposure, Chile

## Abstract

Significant risks to human health have been associated with chronic exposure to low doses of pesticides, a situation which may be frequent among agricultural workers. In this context, and regarding the agricultural-based economy of central Chile, we aimed to explore the genotoxic damage in agricultural workers and reproductive risk among women in rural and urban areas of Curicó, a traditional agricultural district in Chile. Hence, we sampled a group of rural agricultural workers associated with pesticide management (*n* = 30) and an urban unexposed group (*n* = 30). Our results showed that the agricultural workers had higher micronuclei frequencies (MN: β = 13.27; 95% CI _low_ = 11.08, CI _high_ = 15.47) and women had a 40-fold higher risk of reproductive problems (OR = 40.32; 95% CI _low_ = 2.60, CI _high_ = 624.31) than the unexposed group. The factor analysis of mixed data (FAMD) showed that neither the sex nor smoking habits appear to define the ordination of the data. Nevertheless, the exposure level did segregate them in the multidimensional space (explained variance: 35.38% dim-1; 18.63% dim-2). This pilot study highlights the higher risks of biological conditions negatively associated with the health of agricultural workers.

## 1. Introduction

The current volume of agricultural production relies on the extensive use of diverse chemical compounds, among which several are pesticides. However, despite the latter being, by definition, toxic substances, they are deliberately released into the environment to control pests among crops [1,2]. In 2019, more than 4 million tons of these compounds were used in agricultural fields worldwide [3], and due to world population growth, the use of these compounds will continue to increase every year [1]. Undoubtedly, the productive advantages of pesticides are acknowledged. However, their indiscriminate use can be disastrous for both human health and environmental functioning [4], particularly if they are handled and applied in an incorrect way [2].

Concerning human exposure in the agricultural environment, farmers and workers are occupationally exposed to high levels of complex mixtures of pesticides, especially in developing countries [5]. But while the lack or inappropriate use of personal protective equipment can result in severe acute poisoning, prolonged low-level exposure has also a chronic harmful effect on the health of the exposed individuals [2]. Due to the apparent harmlessness of low-dose exposure, it is estimated that many more people are exposed in a low-level and long-term way than those suffering from acute poisoning [6,7]. This becomes more relevant if we consider that an increase in the exposure of the population to pesticides has been projected, due to the pressures imposed by the current climate change dynamics on the agricultural production systems [8], such as enhancing chemical toxicity, increasing rates of chemical degradation, enhancing volatilization of pesticides to the atmosphere or surface deposition of airborne pesticides, or changes in the frequency and amount of pesticides used [9,10].

Among the diverse biological threats of pesticides on human health, their genotoxic potential under low-dose long-term exposures is a major risk factor for several chronic diseases [11], and it has been considered the most studied early effect biomarker [12,13]. Indeed, several epidemiological studies establish a direct relation between occupational exposure to pesticides and cancer [14,15,16,17], diabetes, respiratory, cardiovascular [18,19], autoimmune, and neurodegenerative diseases [20,21]. In addition, a large body of literature has reported the adverse effects of occupational and/or environmental pesticides exposure on reproductive or developmental disorders such as decreased fertility, a higher rate of spontaneous abortions, and congenital malformations [18,22,23,24,25]. In this context, improving the risk estimation of developing a chronic disease seems highly desirable. For this reason, molecular tools like genotoxicity biomarkers, which allow the detection of early effects resulting from the interaction between the individual and pesticides, appear as an important tool for environmental epidemiology [26].

A recent review about the health effects of pesticide exposure in Latin American and the Caribbean (LAC) populations, with a 14% of global agricultural production [27], provided some evidence that exposure to pesticides may adversely impact the health of the populations where the exposure to OP pesticides, carbamates, or to multiple pesticide classes was consistently associated with markers of genotoxicity and adverse neurobehavioral outcomes, particularly among children and farmworkers [13]. In Chile, 21% of the surface of the territory is agricultural land [26]. In the last 25 years, the country has experienced a great agricultural development, consequently, the sales of pesticides increased by almost 30% between 2012 and 2019 [28]. Only in 2019, about 54,450 tons were marketed in the country, being organophosphates, carbamates, and pyrethroids the most commercialized [28]. The Maule region is the area of the country with the largest agricultural exploitation, where 34% of the inhabitants correspond to rural populations in agricultural and livestock occupations [29].

Despite the huge agricultural development in Chile, especially in the Maule region, there are few epidemiological studies which aimed to assess occupational exposure to pesticides in the population and their effects. However, some precedents have already been set where exposed people have higher levels of cognitive impairment [30,31,32], and also, both agricultural workers and the general population of rural areas have a higher risk of developing cancer compared to unexposed population [33].

In the same way, associations between occupational exposure and both genotoxic and/or reproductive health problems have also been established in seasonal women farmers [22,34,35]. Márquez et al. [22] evaluated the cytogenetic damage associated with exposure to mixtures of pesticides through the Micronucleus (MN) assay in 64 agricultural women workers and 30 unexposed women. They found an increased frequency of binucleated cells with micronuclei in the exposed women compared to the unexposed, confirming that occupational exposure to pesticide mixtures could result in an increased cytogenetic damage in this population [22]. Similarly, Zúñiga et al. [34] evaluated the genotoxic damage in 87 females occupationally exposed to pesticides and 54 unexposed women, using the MN and the Sister Chromatid Exchange (SCE) assays, and this study revealed a significant increase in cytogenetic damage in the exposed group. Furthermore, exposure to pesticides was a risk factor for the reproductive health of exposed women for most of the parameters evaluated [34]. Recently, these authors have confirmed the genotoxic effects of pesticide exposure in farmers from Coquimbo region. Additionally, they have reported a genetic susceptibility of this population to metabolize OP pesticides by genotyping PON-1 susceptibility biomarker [35].

Because of the aforementioned, and due to the fact that the Maule region of Chile has the largest proportion of area devoted to agricultural exploitation, it is reasonable to think agricultural workers in this region could be in a constant pesticide exposure. Therefore, in this pilot study, our main objective was to know whether the genotoxic and reproductive risks are increased in a group of agricultural workers exposed to pesticides in the rural area of Curicó city, compared to an urban unexposed group.

## 2. Materials and Methods

### 2.1. Study Design and Population

**Figure 1:**
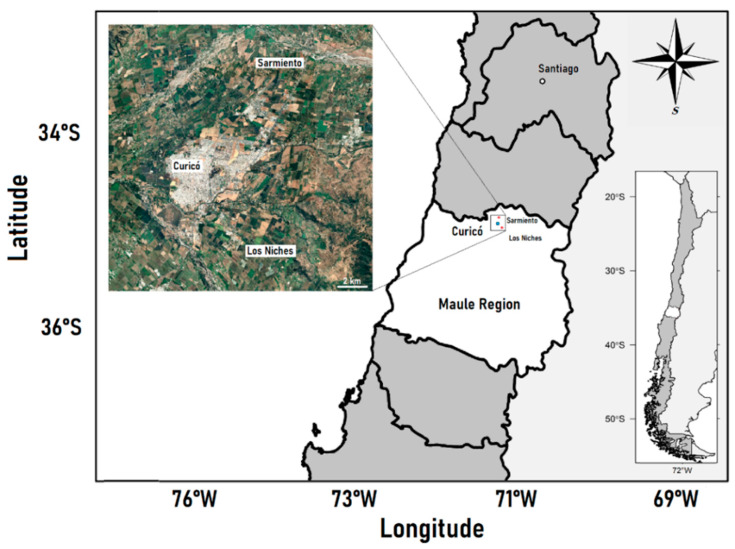
Location of the sampling sites of the study. The main map shows the study site (black square) within Chile and the Maule Region. The inset picture shows the specific sampling points of exposed (Sarmiento and Los Niches) and unexposed (Curicó) groups.

It was a cross sectional study where two groups of adults between 19 and 66 years old from the Maule region (Chile) were conformed: a group of agricultural workers occupationally exposed to pesticides (*n* = 30) from two rural areas of Curicó (Los Niches and Sarmiento), and an urban unexposed group (*n* = 30) from Curicó city (Figure 1). The workers from Sarmiento correspond to men who worked at Los Lirios aerodrome as fumigation pilots, whose occupational exposure occurs through the loading, maintenance and washing of the fumigation planes, and through handling and mixing the pesticides to be used. The workers from Los Niches were seasonal farm workers (men and women) exposed to mixtures of pesticides through terrestrial fumigation, but also by crop and fruit manipulation. The sampling was carried out during the spring-summer season where there is a greater application of pesticides in agriculture. The crops in which the farmworkers worked were mainly fruit trees (such as apples, cherries, and berries).

The unexposed group was composed of men and women who were not associated with agricultural labors and did not have any known exposure to pesticides. All the studied participants were volunteers and signed an informed consent, which was approved along with the study protocol by the Ethic Committee of the Universidad de Concepción.

### 2.2. Fieldwork and Blood Sampling

The participants were previously informed of the study, and after signing the informed consent, the blood samples were obtained. A survey was applied to obtain relevant information such as occupational and demographic records, clinical history, reproductive problems (for women), which include spontaneous abortions, congenital malformations, cases of reproductive problems, and infertility. Five ml of peripheral blood were taken by intravenous puncture in a heparinized vacutainer. The samples were stored at 4 °C and, the next day, they were transported to the Laboratory of Cytogenetics and Toxicological Genetics, Faculty of Biological Sciences of the University de Concepción to conduct the corresponding analysis.

### 2.3. Micronucleus Assay (MN)

This technique was performed according to Fenech [36]. Lymphocytes were cultured from 0.5 mL of blood in a solution containing 4.5 mL of RPMI 1640 culture medium (10% bovine serum fetal, 1% penicillin/streptomycin, 50 μL L-glutamine). The lymphocytes were stimulated with phytohemagglutinin, incubated at 37 °C for 72 h. After 44 h of culturing, Cytochalasin B (6 μg/mL) was added to stop the cytokinesis of the lymphocytes, leaving binucleated cells. After incubation, the lymphocytes underwent brief hypotonic shock (75 mM KCl). Subsequently, the cells were fixed in cold solution of Carnoy (methanol:acetic acid 3:1) freshly prepared. The cell suspension was stored at 4 °C for a minimum of 12 h to ensure complete fixation. Then, two microscope slides per individual were dropped with 2 drops of approximately 20 µL each, stained with 10% Giemsa in 100 mM phosphate buffer, at pH 6.8 for 5 min. The count of the MN was performed according to the criteria of Bolognesi et al. [37]. A thousand binucleated cells were counted in which the presence or absence of MN was scored. Thus, the number of total MN in 1000 binucleated cells was calculated (BNMN). In addition, the nuclear division index (NDI), nucleoplasmic bridges (NPB), and nuclear budding (NBUD) were obtained [36]. The microscopic readings were performed with the coded slides and blinded to avoid bias.

### 2.4. Reproductive Risk

In order to establish the relation between the reproductive problems and pesticides exposure, the information of reproductive history, obtained from women of both groups by means of surveys, was analyzed. A list with the main reproductive disadvantages was made, which were spontaneous abortions, malformations, and infertility [38,39,40].

### 2.5. Statistical Analysis

The analysis performed was focused on both bivariate analyses to compare the level of cytogenetic damage using MN assay between study groups, but also between women who have reported reproductive problems vs. those who have not; and multivariate analysis to explain the level of this genotoxic damage according to the group, regarding confounders.

For the general description of the study population, the *t* Student and Chi square (χ^2^) tests were used to compare continuous and categorical variables, respectively. To compare the level of cytogenetic damage between groups, the *U*–Mann Whitney test was conducted due to their non-parametrical distribution. A linear regression model, adjusted by age, sex and smoking habits was proposed to explain the level of cytogenetic damage. The normal distribution of the fitted model residuals was verified with a Shapiro-Wilks Test. Additionally, to assess the risk of developing reproductive problems among the studied women, a logistic regression model, adjusted by age and smoking habits was conducted. These statistical analyses were performed using the SPSS software v17.0 (SPSS Inc. Chicago, IL, USA). The null hypothesis was rejected when the *p*–value was <0.05.

Finally, to visualize the multidimensional ordination of the evaluated individuals of both unexposed and exposed groups, a factor analysis of mixed data (FAMD), a principal component method which allows the analysis of both quantitative and qualitative variables, was conducted [41]. For the later, in the R-environment v4.2.0 [42] the “FAMD” function from the FactoMineR R-package was used [43].

## 3. Results

### 3.1. Sociodemographic Description

Workers exposed to pesticide mixtures were an average (±SD) age of 39.8 (±12.7) years old, with a range from 23 to 66 years old. Most of them were women (60%), and 56.6% were cigarette smokers. The average working time in contact with pesticides was 9.7 (±9.1) years, where 63.3% were exposed to pesticides for more than 5 years (Table 1). Similarly, in the unexposed group, the average (±SD) age was 39.6 (±12.0) years old with a range from 19 to 62 years old. Most of them were also women (67%), and 20% were cigarette smokers. It can be observed that both groups were similar in age and sex proportions (Table 1). In relation to the clinical history, none of the workers reported having had cancer or having received chemotherapy treatments.

### 3.2. Cytogenetic Damage

**Figure 2:**
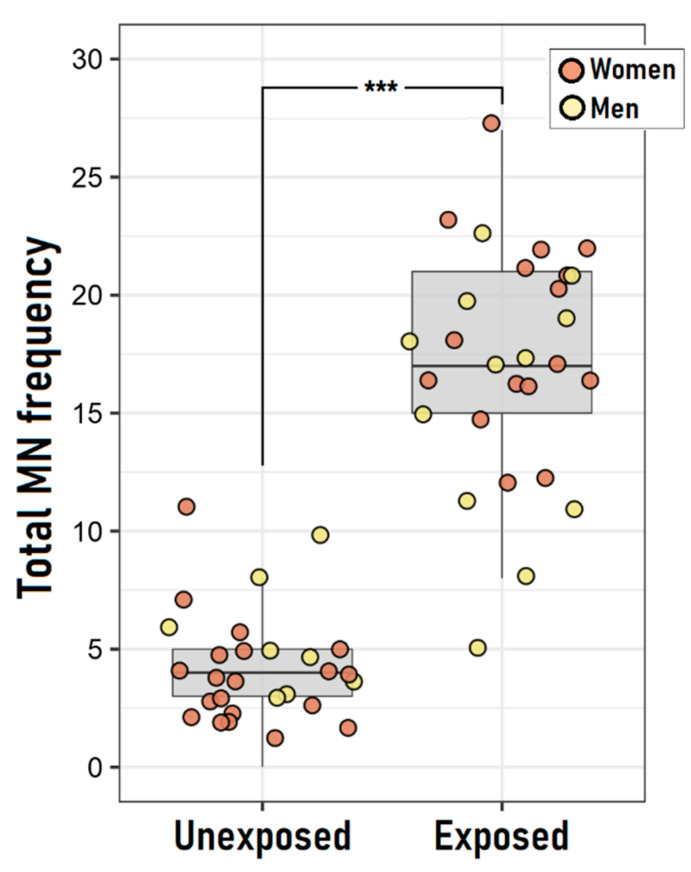
Cytogenetic damage among unexposed and exposed individuals from Curicó (Chile). The boxes and bars represent the interquartile distribution of the individual frequency of binucleated cells with micronuclei (BNMN). The central line inside the boxes represents the data median. Comparison of cytogenetic damage level between study groups was performed through a *U*-Mann Whitney test. *** = *p* < 0.001.

The average frequency of binucleated cells with micronuclei (BNMN) for the unexposed (4.2 ± 2.5) and exposed (17.1 ± 4.9) groups were found to be different, as determined by a *U*-Mann Whitney test (*U* = 12.0, *p* < 0.0001; Figure 2). Additionally, the differences between the frequencies of the nuclear division index (NDI), nucleoplasmic bridge (NPB), and nuclear bud (NBUD) between the unexposed and exposed groups were compared. Increased frequencies of NPB (*p* < 0.001) and NBUD (*p* = 0.002) were also found, but not in the NDI (Table 2).

A linear regression model to estimate the cytogenetic damage variability explained by the exposure group was conducted after being adjusted by age, sex, and level of smoking habits (R^2^ = 0.74; *p* = 0.002) (Table 3). This showed that exposed individuals have higher levels of total MN frequency (β = 13.27; 95% CI: 11.08, 15.47) relative to the unexposed ones. Confounding factors were not significant in explaining the cytogenetic damage level (β_age_ = 0.05; 95% CI: −0.04, 0.13; β_sex_ = 1.09; 95% CI: −1.10, 3.28; β_smoker_ = −0.97; 95% CI: −3.26, 1.31). The normal distribution of the fitted model residuals was verified with a Shapiro-Wilks Test (*p* = 0.2433)

### 3.3. Reproductive Problems and Pesticide Exposure

To evaluate the reproductive risk of the exposed group, the reproductive records such as spontaneous abortions, infertility, and malformations in both groups were analyzed.

Of the sixteen women exposed to pesticide mixtures who responded the reproductive questions (2 did not), it was found that: (i) Three of them had had children with malformations (five children with malformations in total), among which are cleft palate, pilonidal fossa, malformations of the nervous system (such as microcephaly and anencephaly), and Down syndrome (ii) Five women had suffered spontaneous abortions, some of them on more than one occasion (11 abortions in total) and (iii) Two women presented infertility. On the contrary, in women of the unexposed group only one abortion was consigned (Table 2).

**Figure 3:**
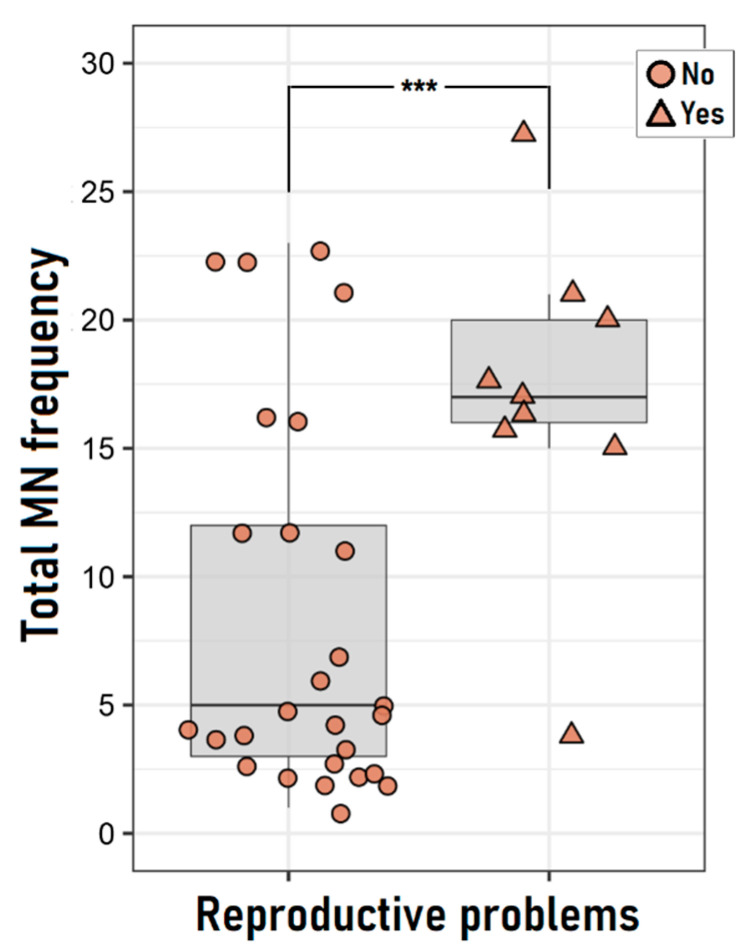
Cytogenetic damage level between women who have reported reproductive problems and those who have not. *** *p* < 0.003 *U*-Mann Whitney test.

Regarding the cytogenetic damage level, women who presented reproductive problems showed higher MN frequency than those without them (Figure 3), probably due to the fact that most of the first one belongs to the exposed group. Following this line, the logistic regression model showed that women belonging to the exposed group have 40-fold increased risk of reproductive problems than those belonging to the unexposed group (OR = 40.32; 95% CI: 2.60, 624.31). It’s important to note that among exposed women, similar MN frequency between those who have reported reproductive problems and those who have not was seen (Mann Whitney test, *p* = 0.730).

**Figure 4:**
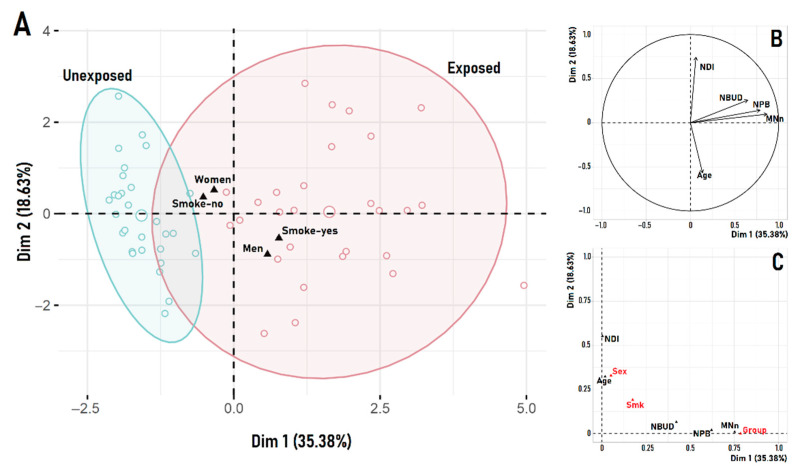
Results from the factor analysis of mixed data (FAMD). It shows the similarity relations between samples (**A**), the correlation circle for continuous variables (**B**) and the relation among all variables in the bidimensional space (**C**). Black triangles in (**A**) show the centroid for the respective group category. For the exposure groups variable, the red-blue ellipses denote the 95% confidence interval around the group (unexposed, exposed) centroid, which corresponds to the empty circle (slightly larger than the rest) within the respective ellipse. Red names in (**C**) correspond to categorical variables, while those in black represent continuous variables. MN: Micronucleus frequency; NDI: nuclear division index; NPB: nucleoplasmic bridge; NBUD: nuclear bud.

This significant influence of the exposure groups on the sampled variables is clearly observed in the FAMD ordination (Figure 4). In this analysis, besides the cytogenetic (MN, NDI, NBUD, NPB) and grouping variables (unexposed, exposed), the modeled covariables (age, sex, smoking habits) were also included. However, most of the explained variability corresponds to the exposure groups, which dominates the first dimension (35.38% of explained variability) of the FAMD (Figure 4B). Accordingly, most genotoxic variables (MN, NPB, NBUD), appear associated to it (Figure 4C).

## 4. Discussion

Pesticides are widely used in the world for pest control, especially in agriculture. Due to its high biological activity and its long persistence in the environment, many of these compounds affect non-targeted organisms, and humans are not the exception [2]. This occurs mainly through the occupational exposure to pesticides among workers involved in its manufacture, and users in the agricultural sector such as agricultural workers and professional pesticide applicators [44]. However, since the agricultural activity is transversal to human societies around the globe, there is great concern about the long-term impact of pesticides on human health.

The results presented in this pilot study in the Maule region, Chile, showed that among the sampled population, the group of agricultural workers exhibit a high rate of genotoxic damage (MN, NPB, and NBUD frequencies) relative to their control. The results are consistent with other genotoxic studies conducted in Chile, but also from Latin American [45] and other developing countries with a similar productive model and lack of regulatory policies. For example, Kahl et al. [46] evaluated genotoxic damage in 121 tobacco field workers exposed to complex pesticides mixtures in Brazil. For this study, they used the Comet and Micronucleus assays, finding significantly increased DNA damage when comparing exposed and unexposed populations [46]. Another study conducted in Mexico, in three agricultural rural communities, where DNA damage was evaluated by Comet and Micronuclei assays in 111 agricultural workers and 60 unexposed individuals, showed that DNA tail migration and MN frequency increased significantly in the exposed group [47]. A cross-sectional study among paddy farmers in Malaysia suggests that farmers who are chronically exposure to a mixture of organophosphates (*n* = 160) have at least 2-fold increase of DNA damage as compared with the control group (*n* = 160) [48].

In relation to the Chilean studies, in the Bío-Bío Region, an area with intensive use of agricultural pesticides, an increase in the frequency of MN of 3.7-fold was found in a group of female agricultural compared to the respective unexposed group [22]. Accordingly, the authors conclude that since these workers performed the thinning, pruning, and harvesting of the local fruit crops, their exposure to pesticides might be the cause behind the observed selective genotoxicity.

In Chile, not only genotoxic and reproductive effects associated to pesticide exposure have been demonstrated, but also neurotoxicity, cancer, and other general health problems [49]. The regulation of the application of pesticides is very recent, and only the minimum health and safety standards for agricultural workers are met [49]. This is due to the fact that, in many cases, they do not use personal protection elements and, generally, the clothes they use at work are the same with which they go home, indirectly exposing their families. In addition, the re-entry period (minimum time they must wait after the application of pesticides for the entry of people or animals to the treated area) is often not respected, which makes the exposure even greater [22].

When analyzing the confounding factors (gender and smoking habits) no differences were found, so for the bioassay conducted in this work, the differences found between the unexposed and exposed groups allow us to suggest that there are alterations in the genetic material of agricultural workers who handle and apply pesticides, manipulated crops and fruit, or loaded and washed the plains for aerial applications.

Globally, the agricultural worker population is composed of an increasing number of women [50]. Pesticides, mainly organochlorines, organophosphates, pyrethroids, and triazines can have a harmful impact on human fertility [51]. It has been reported that women engaged in agriculture are more susceptible to reproductive disorders, such as menstrual disturbances, infertility (more than 12 months of trying without pregnancy), premature labor, spontaneous abortion, fetal death, congenital malformations, and children with low birth weight [51,52]. The genotoxic damage found in exposed women of this study was associated with reproductive problems such as malformations, spontaneous abortions, and infertility; in other words, the women who have declared reproductive problems belonged to the exposed group.

Various studies report an increased incidence of these reproductive problems because of pesticide exposure in female agricultural workers. Rahimi et al. [52] studied 308 women between the ages of 16 and 49 who worked in greenhouses and 338 unexposed controls from nearby villages in southern Iran. The data obtained revealed that the rate of spontaneous abortions, infertility, low birth weight, abnormal births and premature births were significantly higher among greenhouse workers exposed to pesticides compared to the control group [52]. In the Bío-Bío Region, Chile, a similar study was conducted using the MN and SCE assays in 87 exposed and 54 unexposed women, where higher levels of cytogenetic damage were found in the exposed group for both trials. In that study, the exposure to pesticides was also seen as a risk factor for the reproductive health of exposed women [34].

### Limitations of the Study

Although we do not know the pesticides to which the workers were specifically exposed, because we did not evaluate the exposure sources neither routes, Cortés et al. [26] carried out a passive sampling of aerial pesticides in the town of Molina, 21 km from our sampling site, where 9 pesticides were detected. The most abundant was chlorpyrifos, and diazinon, atrazine, dimethoate, metolachlor, simazine, terbuthylazine, and tebuconazole were also detected [26]. These results were similar to those reported in other areas of the country [16,31]. However, there is a recent study which has reported local and regional sources of organochlorine pesticides (OCP) in a rural zone in central Chile, and concluded that soil volatilization is the major OCP source [53]. This family of pesticides has also been associated with cytogenetic damages [54], cryptorchidism [55], fertility [56], and reproductive hormone disruptions [57,58]. In our study, the infertility has not been medically diagnosed, it was only assessed by each woman participating in the study as the years she had been trying to get pregnant.

Another limitation of this study is that other forms of exposure such as dermal and ingestion are not considered. These are other important routes of pesticide entry into the body, which were not evaluated in this study. In addition, we have not directly measured the levels of pesticides and their metabolites in the study group, demonstrating pesticide exposure in an indirect way.

Since our sample size is small, more research is needed to provide evidence to contribute to a robust surveillance system in this area of the Maule region, thus improving public policies.

## 5. Conclusions

The results of this pilot study tend toward confirming the genotoxic effects of pesticide exposure in farmworkers, also suggesting that the occupational exposure may constitute a risk factor for the reproductive health of women of childbearing age. In this regard, it is relevant to highlight the request to make decisions to protect the health of the rural population, through the development of public policies based on local evidence.

## Figures and Tables

**Table 1:** General characteristics of study population.

*Sociodemographic*	Unexposed (*n* = 30)	Exposed (*n* = 30)	*p*-Value
Age (years ± SD) ^a^	39.6 ± 12.0	39.8 ± 12.7	0.93
Exposure time (years ± SD)	-	9.7 ± 9.1	-
Sex ^b^			
Men (n, %)	10 (33)	12 (40)	0.40
Women (n, %)	20 (67)	18 (60)	
Smoke habit ^b^			
No (n, %)	22 (73)	13 (43)	0.02
Yes (n, %)	8 (27)	17 (57)	

^a^: *t*-Student; ^b^: Independence χ^2^.

**Table 2:** Cytogenetic damage levels and frequency of reproductive problems between groups.

**Cytogenetic Damage ^a^**	**Unexposed** **(*n* = 30)**	**Exposed** **(*n* = 30)**	***p*-Value**
Total MN	4.2 ± 2.5	17.1 ± 4.9	<0.001
NDI	1.9 ± 0.1	1.9 ± 0.1	0.690
NPB	0.1 ± 0.5	1.3 ± 1.2	<0.001
NBUD	0.1 ± 0.3	0.6 ± 0.9	0.002
**Reproductive Problems ^b,c^**	**Unexposed** **(*n* = 20)**	**Exposed** **(*n* = 16)**	***p*-Value**
Women with reproductive problems	1 (5.0)	8 (50)	0.003
Women with spontaneous abortion	1 (5.0)	5 (31.3)	0.049
Women with children with malformation	0 (0)	3 (18.8)	0.078
Infertility	0 (0)	2 (12.5)	0.190

MN: Micronucleus frequency; NDI: nuclear division index; NPB: nucleoplasmic bridge; NBUD: nuclear bud. ^a^ Values are expressed as mean ± SD. *p*-values were calculated using U-Mann Whitney test; ^b^ Values are expressed as frequencies (%). *p*-values were calculated using independence χ^2^; ^c^ analysis based on women only (*n* = 36).

**Table 3:** Multiple linear regression model to estimate cytogenetic damage in function of the exposure, adjusted by confounders.

Explanatory Variables	Coefficient			Model		
	β	95% CI	*p*-Value	R^2^	Adjusted R^2^	*p*-Value
Group	13.27	11.08–15.470	<0.001	0.75	0.74	0.002
Age	0.05	−0.04–0.13	0.279			
Sex	1.09	−1.10–3.28	0.321			
Smoker	−0.97	−3.26–1.31	0.397			

Reference group for categorical variables: Unexposed = 0, Exposed = 1; Men = 0, Women = 1; non-smokers = 0, smokers = 1.

## Data Availability

Not applicable.

## References

[1] Kim K.H., Kabir E., Jahan S.A. (2017). Exposure to pesticides and the associated human health effects. Sci. Total Environ..

[2] Damalas C.A., Koutroubas S.D. (2016). Farmers’ Exposure to Pesticides: Toxicity Types and Ways of Prevention. Toxics.

[3] Maggi F., Tang F.H.M., la Cecilia D., McBratney A. (2019). PEST-CHEMGRIDS, global gridded maps of the top 20 crop-specific pesticide application rates from 2015 to 2025. Sci. Data.

[4] Jepson P.C., Murray K., Bach O., Bonilla M.A., Neumeister L. (2020). Selection of pesticides to reduce human and environmental health risks: A global guideline and minimum pesticides list. Lancet Planet. Health.

[5] Hutter H.P., Poteser M., Lemmerer K., Wallner P., Sanavi S.S., Kundi M., Moshammer H., Weitensfelder L. (2020). Indicators of Genotoxicity in Farmers and Laborers of Ecological and Conventional Banana Plantations in Ecuador. Int. J. Environ. Res. Public Health.

[6] Ross S.M., McManus I.C., Harrison V., Mason O. (2013). Neurobehavioral problems following low-level exposure to organophosphate pesticides: A systematic and meta-analytic review. Crit. Rev. Toxicol..

[7] Brender J.D., Maantay J.A., Chakraborty J. (2011). Residential proximity to environmental hazards and adverse health outcomes. Am. J. Public Health.

[8] Gatto M.P., Cabella R., Gherardi M. (2016). Climate change: The potential impact on occupational exposure to pesticides. Ann. Ist. Super. Sanita.

[9] Noyes P.D., McElwee M., Miller H.D., Clark B.W., Van Tiem L.A., Walcott K.C., Erwin K.N., Levin E.D. (2009). The toxicology of climate change: Environmental contaminants in a warming world. Environ. Int..

[10] Weiss F.T., Leuzinger M., Zurbrügg C., Eggen H.I. (2016). Chemical Pollution in Low- and Middle-Income Countries.

[11] Bolognesi C., Creus A., Ostrosky-Wegman P., Marcos R. (2011). Micronuclei and pesticide exposure. Mutagenesis.

[12] Ladeira C., Smajdova L. (2017). The use of genotoxicity biomarkers in molecular epidemiology: Applications in environmental, occupational and dietary studies. AIMS Genet..

[13] Zúñiga-Venegas L.A., Hyland C., Muñoz-Quezada M.T., Quirós-Alcalá L., Butinof M., Buralli R., Cardenas A., Fernandez R.A., Foerster C., Gouveia N. (2022). Health Effects of Pesticide Exposure in Latin American and the Caribbean Populations: A Scoping Review. Environ. Health Perspect..

[14] Turner M.C., Wigle D.T., Krewski D. (2011). Residential pesticides and childhood leukemia: A systematic review and meta-analysis. Cien. Saude Colet..

[15] Van Maele-Fabry G., Libotte V., Willems J., Lison D. (2006). Review and meta-analysis of risk estimates for prostate cancer in pesticide manufacturing workers. Cancer Causes Control.

[16] Weichenthal S., Moase C., Chan P. (2012). A review of pesticide exposure and cancer incidence in the agricultural health study cohort. Cien. Saude Colet..

[17] Burns C.J., Juberg D.R. (2021). Cancer and occupational exposure to pesticides: An umbrella review. Int. Arch. Occup. Environ. Health.

[18] Mostafalou S., Abdollahi M. (2013). Pesticides and human chronic diseases: Evidences, mechanisms, and perspectives. Toxicol. Appl. Pharmacol..

[19] Czajka M., Matysiak-Kucharek M., Jodłowska-Jędrych B., Sawicki K., Fal B., Drop B., Kruszewski M., Kapka-Skrzypczak L. (2019). Organophosphorus pesticides can influence the development of obesity and type 2 diabetes with concomitant metabolic changes. Environ. Res..

[20] Singh C., Ahmad I., Kumar A. (2007). Pesticides and metals induced Parkinson’s disease: Involvement of free radicals and oxidative stress. Cell Mol. Biol. (Noisy-le-grand).

[21] Song C., Kanthasamy A., Anantharam V., Sun F., Kanthasamy A.G. (2010). Environmental neurotoxic pesticide increases histone acetylation to promote apoptosis in dopaminergic neuronal cells: Relevance to epigenetic mechanisms of neurodegeneration. Mol. Pharmacol..

[22] Márquez C., Villalobos C., Poblete S., Villalobos E., de Los Angeles García M., Duk S. (2005). Cytogenetic damage in female Chilean agricultural workers exposed to mixtures of pesticides. Environ. Mol. Mutagen..

[23] Snijder C.A., Brouwers M.M., Jaddoe V.W., Hofman A., Roeleveld N., Burdorf A. (2011). Occupational exposure to endocrine disruptors and time to pregnancy among couples in a large birth cohort study: The Generation R Study. Fertil. Steril..

[24] Kumar S., Sharma A., Kshetrimayum C. (2019). Environmental & occupational exposure & female reproductive dysfunction. Indian J. Med. Res..

[25] Roberts T.C. (2014). The MicroRNA Biology of the Mammalian Nucleus. Mol. Ther.-Nucleic Acids.

[26] Cortes S., Pozo K., Llanos Y., Martinez N., Foerster C., Leiva C., Ustáriz J., Přibylová P., Klánová J., Jorquera H. (2020). First measurement of human exposure to current use pesticides (CUPs) in the atmosphere of central Chile: The case study of Mauco cohort. Atmos. Pollut. Res..

[27] OECD/FAO (Organization for Economic Cooperation and Development, F.A.O.) (2019). AOCED-FAO Agricultural Outlook 2019–2028. Special Focus: Latin America.

[28] Servicio Agricola y Ganadaro (SAG) (2019). División Protección Agrícola y Forestal. Departamento Regulación y Control de Insumos Silvoagrícolas Subdepartamento de Plaguicidas y Fertilizantes. Ministerio de Agricultura. *Declaración De Ventas De Plaguicidas De Uso Agrícola Año 2019*; SAG. Santiago de Chile. https://www.sag.gob.cl/sites/default/files/declaracion_de_ventas_de_plaguicidas_ano_2019_0.pdf.

[29] Muñoz-Quezada M.T., Lucero B., Iglesias V., Muñoz M.P. (2014). Exposure pathways to pesticides in schoolchildren in the Province of Talca, Chile. Gac. Sanit..

[30] Ramírez-Santana M., Farías-Gómez C., Zúñiga-Venegas L., Sandoval R., Roeleveld N., Van der Velden K., Scheepers P.T.J., Pancetti F. (2018). Biomonitoring of blood cholinesterases and acylpeptide hydrolase activities in rural inhabitants exposed to pesticides in the Coquimbo Region of Chile. PLoS ONE.

[31] Ramírez-Santana M., Zúñiga-Venegas L., Corral S., Roeleveld N., Groenewoud H., van der Velden K., Scheepers P.T.J., Pancetti F. (2020). Association between cholinesterase’s inhibition and cognitive impairment: A basis for prevention policies of environmental pollution by organophosphate and carbamate pesticides in Chile. Environ. Res..

[32] Muñoz-Quezada M.T., Lucero B., Iglesias V., Levy K., Muñoz M.P., Achú E., Cornejo C., Concha C., Brito A.M., Villalobos M. (2017). Exposure to organophosphate (OP) pesticides and health conditions in agricultural and non-agricultural workers from Maule, Chile. Int. J. Environ. Health Res..

[33] Cabello G., Valenzuela-Estrada M., Siques P., Brito J., Parra E., Valdivia U., Lavin C., Manríquez A., Ortega A. (2013). Relation of Breast Cancer and Malathion Aerial Spraying in Arica, Chile. Int. J. Morphol..

[34] Venegas L.A.Z., Urrizola C.G.M., Palacios M.S.D. (2007). Estudio citogenético y reproductivo en mujeres temporeras expuestas a pesticidas de la Vlll Región de Chile. Theoria.

[35] Zúñiga-Venegas L., Pancetti F.C. (2022). DNA damage in a Chilean population exposed to pesticides and its association with PON1 (Q192R and L55M) susceptibility biomarker. Environ. Mol. Mutagen..

[36] Fenech M. (2007). Cytokinesis-block micronucleus cytome assay. Nat. Protoc..

[37] Bolognesi C., Knasmueller S., Nersesyan A., Thomas P., Fenech M. (2013). The HUMNxl scoring criteria for different cell types and nuclear anomalies in the buccal micronucleus cytome assay—An update and expanded photogallery. Mutat. Res..

[38] Skakkebæk N.E., Lindahl-Jacobsen R., Levine H., Andersson A.M., Jørgensen N., Main K.M., Lidegaard Ø., Priskorn L., Holmboe S.A., Bräuner E.V. (2022). Environmental factors in declining human fertility. Nat. Rev. Endocrinol..

[39] Medina-Carrilo L., Rivas-Solis F., Fernández-Argüelles R. (2002). Risk for congenital malformations in pregnant women exposed to pesticides in the state od Nayarit, Mexico. Ginecol. Obstet. Mex..

[40] Baldacci S., Gorini F., Santoro M., Pierini A., Minichilli F., Bianchi F. (2018). Environmental and individual exposure and the risk of congenital anomalies: A review of recent epidemiological evidence. Epidemiol. Prev..

[41] Pagès J. (2004). Analyse Factorielle de Donnees Mixtes. Revue. Stat. Appl..

[42] R Core Team (2022). R: A Language and Environment for Statistical Computing.

[43] Lê S., Josse J., Husson F. (2008). FactoMineR: An R Package for Multivariate Analysis. FactoMineR: An R Package for Multivariate Analysis. J. Stat. Softw..

[44] Ye M., Beach J., Martin J.W., Senthilselvan A. (2013). Occupational pesticide exposures and respiratory health. Int. J. Environ. Res. Public Health.

[45] Zúñiga-Venegas L., Muñoz-Quezada M.T., Hyland C., Butinof M., Calaf G., Handal A.J., Quirós-Alcalá L., Cortés S., Mora A.M. (2022). Pesticide exposure and health effects in Latin American and the Caribbean populations: A systematic review. Environ. Health Perspect..

[46] Kahl V.F.S., da Silva F.R., Alves J.D.S., da Silva G.F., Picinini J., Dhillon V.S., Fenech M., de Souza M.R., Dias J.F., de Souza C.T. (2018). Role of PON1, SOD2, OGG1, XRCC1, and XRCC4 polymorphisms on modulation of DNA damage in workers occupationally exposed to pesticides. Ecotoxicol. Environ. Saf..

[47] Carbajal-López Y., Gómez-Arroyo S., Villalobos-Pietrini R., Calderón-Segura M.E., Martínez-Arroyo A. (2016). Biomonitoring of agricultural workers exposed to pesticide mixtures in Guerrero state, Mexico, with comet assay and micronucleus test. Environ. Sci. Pollut Res..

[48] How V., Hashim Z., Ismail P., Omar D., Said S.M., Tamrin S.B. (2015). Characterization of risk factors for DNA damage among paddy farm worker exposed to mixtures of organophosphates. Arch. Environ. Occup. Health.

[49] Zúñiga-Venegas L., Saracini C., Pancetti F., Muñoz-Quezada M.T., Lucero B., Foerster C., Cortés S. (2021). Pesticide exposure in Chile and population health: Urgency for decision making. Gac. Sanit..

[50] Doss C.R. (2018). Women and agricultural productivity: Reframing the Issues. Dev. Policy Rev..

[51] Fucic A., Duca R.C., Galea K.S., Maric T., Garcia K., Bloom M.S., Andersen H.R., Vena J.E. (2021). Reproductive Health Risks Associated with Occupational and Environmental Exposure to Pesticides. Int. J. Environ. Res. Public Health.

[52] Rahimi T., Rafati F., Sharifi H., Seyedi F. (2020). General and reproductive health outcomes among female greenhouse workers: A comparative study. BMC Women’s Health.

[53] Llanos Y., Cortés S., Martínez A., Pozo K., Přibylová P., Klánová J., Jorquera H. (2022). Local and regional sources of organochlorine pesticides in a rural zone in central Chile. Atmos. Pollut. Res..

[54] Anguiano-Vega G.A., Cazares-Ramirez L.H., Osten J.R.-V., Santillan-Sidon A.P., Vazquez-Boucard C.G. (2020). Risk of genotoxic damage in schoolchildren exposed to organochloride pesticides. Sci. Rep..

[55] Montes L.P.B., Waliszewski S., Hernández-Valero M., Sanín-Aguirre L., Infanzón-Ruiz R.M., Jañas A.G. (2010). Prenatal exposure to organochlorine pesticides and cryptorchidism. Cien. Saude Colet..

[56] Bastos A.M., Souza M.O.C., Filho G.L.A., Krauss T.M., Pavesi T., Silva L.E. (2013). Organochlorine compound levels in fertile and infertile women from Rio de Janeiro, Brazil. Arq. Bras. Endocrinol. Metabol..

[57] Blanco-Muñoz J., Lacasaña M., Aguilar-Garduño C., Rodríguez-Barranco M., Bassol S., Cebrián M.E., López-Flores I., Ruiz-Pérez I. (2012). Effect of exposure to p, p′-DDE on male hormone profile in Mexican flower growers. Occup. Environ. Med..

[58] Freire C., Koifman R.J., Sarcinelli P.N., Rosa A.C., Clapauch R., Koifman S. (2014). Association between serum levels of organochlorine pesticides and sex hormones in adults living in a heavily contaminated area in Brazil. Int. J. Hyg. Environ. Health.

